# Regular soccer training improves pulmonary diffusion capacity in 6 to 10 year old boys

**DOI:** 10.1186/s13102-023-00757-6

**Published:** 2023-11-02

**Authors:** Rim Dridi, Nadia Dridi, Nabil Gmada, Ismail Laher, Ayoub Saeidi, Urs Granacher, Hassane Zouhal

**Affiliations:** 1 Research Laboratory LR23JS01 (Sport, Performance, Health and Society, Higher Institute of Sport and Physical Education of Ksar Said, University of La Manouba, Tunis, 2010 Tunisia; 2 Physical Education and Sport Sciences Department, Sultan Qaboos University, Muscat 123, Oman; 3 Department of Anesthesiology, Pharmacology, and Therapeutics, Faculty of Medicine, University of British Columbia, Vancouver, Canada; 4 Department of Physical Education and Sport Sciences, Faculty of Humanities and Social Sciences, University of Kurdistan, Sanandaj, Kurdistan Iran; 5 Department of Sport and Sport Science, Exercise and Human Movement Science, University of Freiburg, Freiburg, Germany; 6 Univ Rennes, M2S (Laboratoire Mouvement, Sport, Rennes, Santé EA 1274, F-35000 France; 7 Institut International des Sciences du Sport (2I2S), Irodouer, 35850 France

**Keywords:** Capillary blood volume, Alveolar capillary membrane diffusing capacity, Football, Children, Exercise

## Abstract

**Background:**

Soccer is one of the most attractive sports around the globe for children and adolescents, and the benefits of soccer training are often shown. Due to the intermittent character of soccer with random changes between high-intensity activity and low-intensity play, athletes’ aerobic (respiratory) capacity is specifically stimulated. However, little is known about the effects of regular soccer practice on pulmonary diffusion capacity (TL) in young players, even though it is the most popular sport in the world.

**Objectives:**

To analyze the effects of 28 weeks of regular soccer training versus a non-activity control period on the TL, the alveolar-capillary membrane diffusion capacity (DM) as well as the capillary blood volume (Vc) in healthy prepubertal boys aged 6 to 10 years.

**Methods:**

For this purpose, boys were randomly assigned to a soccer training group (SG, n = 40) or a control group (CG, n = 40). Pre and post-intervention, all participants performed an all-out graded bicycle ergometer test to measure maximal oxygen uptake (VO_2max_) and maximal aerobic power (MAP). A respiratory maneuver was performed at rest and just at the end of the test to measure the TL for carbon monoxide (TL_CO_) and nitric oxide (TL_NO_), DM, as well as Vc.

**Results:**

There were no significant baseline between-group differences for any of the assessed parameters (p > 0.05). Significant group-by-time interactions were found for most pulmonary parameters measured at rest (p < 0.05), with effect size (ES) values ranging from small-to-large (0.2 < ES < 4.0), except for VA (p = 0.3, ES = 0.006). Post-hoc tests indicated significant DM (p < 0.05; 0.2 < ES < 4.0), TL_NO_ (p < 0.01; 0.22 < ES < 4.0), TL_CO_ (p < 0,01; 0.24 < ES < 4.0) and Vc (p = 0.01; 0.404 < ES < 0.6) improvements for SG but not CG. Significant group-by-time effects were identified for HRmax and VO_2_max (p < 0.001; ES = 0.5 and p = 0.005; ES = 0.23 respectively). The post-hoc analyses indicated a significant decrease in HRmax and a significant increase in VO_2_max in the SG (p < 0.001; ES = 0.5 and p = 0.005, ES = 0.23, respectively) but not in CG. Values for TL_CO_ increased by almost 20%; Vc of 14% DM of 8% and VA of 10% at the end of maximal exercise in SG. Furthermore, the percentage improvement was less notable in the control group (7.5% for TL_CO_; 2% for Vc; 5% for DM and 4% for VA).

**Conclusion:**

Regular soccer training significantly improves pulmonary vascular function and increases DM and Vc after exercise in prepubertal boys. The observed adaptations are most likely due to better recruitment of additional pulmonary capillary function. However, the stepwise linear regression analyses indicated that increases in pulmonary vascular function were not related to improvements in VO_2max_ and MAP.

**Supplementary Information:**

The online version contains supplementary material available at 10.1186/s13102-023-00757-6.

## Introduction

In youth, soccer is one of the most attractive sports around the globe, and the benefits of soccer training have previously been shown in the form of gains in muscle mass [1], and improved cardiovascular [2] and body fat profiles [3] in youth and adults. The available scientific literature is uniform and shows the positive effects of soccer training in youth on physical and cardiac parameters: Franca et al. 2023 [4] reported that young soccer players were significantly taller and heavier than controls at baseline and follow-up. McMillan et al. 2005 [5] pointed out that soccer specfic endurance training in youth soccer players should increase VO_2_max by increasing the maximal cardiac output by improving stroke volume. Furthermore, youth soccer is characterized by constant changes in the game intensity due to a variety of activities involving walking, running and sprinting with changes of direction, as well as jumping with or without the ball and/or opponents [6, 7]. Although extensive research is available in youth soccer on mesure of physical fitness [4], only few studies sought to assess the benefits of regular participation in soccer training on pulmonary diffusing capacity in pre-puberal (under 10 years of age). Of note, this research question has previously been examined in pediatric patients [8] and in different youth sports such as basketball or running (middle distance runners) [9].

This is supported by findings showing that regular practice of an intermittent sport improves alveolar diffusion in children [10], while high-intensity intermittent efforts increase ventilatory performance [11], suggesting that intermittent exercise (i) improves both respiratory and cardiac demand [12] and (ii) enhances the distensibility of the pulmonary capillary bed.

The assessment of pulmonary diffusion capacity for carbon monoxide and nitric oxide (TL_CO/NO_) is widely used to estimate the alveolar-capillary structure [13–16], and is related to two variables: the membrane factor (DM) and the pulmonary capillary volume (Vc) [17]. Different researchers investigated the effects of sports practice such as basketball [10], middle-distance running [9], or swimming [18] on the vascularization of the pulmonary capillary bed and the distensibility of the lung surface [9, 19].

Although the improvement of the functional parameters; such as maximum VO_2_max and maximum aerobic power; after regular soccer training, has been shown in previous studies [7, 20], the effect of soccer practice on the behavior of the pulmonary capillary bed in children remains unknown. This prompted us to evaluate the effects of soccer training versus a control on the transfer capacity of nitric oxide (NO) and carbon monoxide (CO) in healthy prepubertal boys by measuring the alveolar volume, the pulmonary capillary volume, the membrane, and the pulmonary diffusing capacity at rest and directly after completion of a graded all-out test using the TL_NO/CO_ double transfer method. We hypothesized that soccer training (i) could improve lung function associated with increased pulmonary vascular development [7], and (ii) may produce increases in TL_CO_, Vc, and DMco in prepubertal boys [7, 10].

## Methods

### Participants

The required sample size was calculated with G*Power (Version 3.1, University of Dusseldorf, Germany) [21]. The a priori power analysis was computed with an assumed power of 0.90, an alpha level of 0.01, and an effect size of Cohen’s f = 0.26 for the pulmonary diffusion capacity (TL) [10]. The analysis revealed that a total sample size of N = 60 would be sufficient to detect a significant group-by-time interaction effect. Accordingly, 80 participants were enrolled to account for potential dropouts due to injuries.

Eighty healthy prepubertal boys aged 6 to 10 years, with normal lung volumes and regular flow-volume curves and no history of cardiopulmonary disease or allergies volunteered to participate in the study. A respiratory functional exploration was carried out by a medical doctor including a health questionnaire to screen the medical history of the participants. The boys were randomly assigned into two groups:

- The soccer group (SG, n = 40) participated in four weekly soccer sessions, each lasting 75 min over a period of 28 weeks.
- The control group (CG, n = 40) matched according to age, body mass, and height with the SG participated only in regular physical education lessons without any other extracurricular sports activities.

All participating boys attended the same school located near the soccer training center. After written and oral information was provided on the risks and benefits of the study, written informed consent for study participation was obtained from all study participants and their parents or legal guardians before the study started. The ethics committee of the Sousse Medical University (Tunisia) approved the study. The study was conducted in accordance with the latest version of the Declaration of Helsinki. The physical characteristics of the study participants at the time of inclusion are listed in Table 1.

**Table 1:** Participant characteristics and maximal exercise performances of the participating boys before the start of the study (pre: T1) and after completion of the study (post: T_2_)

Parameters	Soccer training group (Age = 8 ± 2years; N = 40)			Control group (Age = 8 ± 2years; N = 40)			Analysis of variance (ANOVA) and effect sizes (eta-squared)					
							Group		Time		Group x Time	
	Pre	Post	ES	Pre	Post	ES	p	η2	p	η2	p	η2
Body mass (kg)	38 ± 3	39.5 ± 3	0.43	39 ± 8	39.5 ± 5	0.06	0.73	0.001	0.31	0.007	0.66	0.001
Height (cm)	148 ± 3	158 ± 2	4.02	147 ± 6	156 ± 5	1.65	0.03	0.03	0.00	0.56	0.54	0.002
Resting HR (bpm)	88 ± 5	83 ± 4	0.71	89 ± 4	90 ± 6	0.24	0.02	0.15	0.08	0.20	0.40	0 0.80
Maximal heart rate (bpm)	199 ± 2	194 ± 2	1.37	196 ± 3	196 ± 4	0.18	0.0001	0.33	0.001	0.44	0.0001	0.50
VO_2_max (ml·min^− 1^·kg^− 1^)	34.8 ± 1.3	36 ± 1.4	1.30	34 ± 0.6	34.9 ± 0.9	0.11	0.03	0.14	0.002	0.26	0.005	0.23
Maximum Aerobic Power (Watts)	130 ± 2	135 ± 4	1.48	129 ± 2	132 ± 2	1.45	0.0001	0.10	0.0001	0.35	0.04	0.25

Data are mean ± standard deviation (SD).

### Procedures

Baseline (***Test 1***) anthropometric data (body height to the nearest 0.1 cm and body mass to the nearest 100 g) were collected using standard stadiometers (Seca™, Hamburg, Germany) and scales (Tefal, France). Maximal oxygen consumption (VO_2_max) and maximal aerobic power (MAP) were determined using standard protocols with the all-out tests performed on a bicycle ergometer (Monark cycle). The cycle ergometer was chosen for security reasons as the participants are boys aged 6 to 10 years and also the treadmill is more stressful. The participants performed unloaded cycling at 60–65 revolutions/min (rpm) for the first minute, after which the work rate was increased every minute according to the Cooper and Weiler-Ravell procedure [22] until VO_2_max was reached. The examiners performing the exercise test were blinded for group allocation.

Oxygen consumption (VO_2_) and carbon dioxide (VCO_2_) production were determined using a calibrated metabolic measurement system (MedGraphics CPX St Paul, MN, USA). The transfers of nitric oxide (NO) and carbon monoxide (CO) were measured simultaneously during a single breath maneuver using an automated apparatus (Medisoft, Dinant, Namur, Belgium), following the latest ERS Guidelines [23]. Each participant performed three validated transfer measurements [9, 10]: two at rest (before exercise) and another at the end of the maximal exercise test. After the test was completed, participants remained seated on the bicycle ergometer. An examiner fixed the nose clip and the mouthpiece to begin the respiratory maneuver under the guidance of the examiner. Participants were informed about the importance of test standardization to achieve better test reproducibility. Test validity was visually checked by examining the trace depicting volume changes during the maneuver. In other words, the computer-generated trace should lack a pause during the fast inspiration, be flat during the breath hold, and be continuous during expiration. The test was considered valid if these criteria were met. All participants received three familiarization trials to practice the maneuvers.

DM and Vc were determined from TL_NO_ and TL_CO_ values as were previously described by Dridi et al. [9]. Since the reactivity of NO and hemoglobin was considered very high and its inverse negligible, TL_NO_ was considered equivalent to DM_NO_. DM_CO_ was determined using the coefficient of proportionality (a) and the DM values of the two gases (aDM_NO_ = aDM_CO_ = 1.97) following “Graham’s law.

The same tests (maximal O_2_ consumption, maximal aerobic power, and NO/CO transfer) were repeated 28 weeks later ***(Test 2)***. All tests and exercise measurements were performed on the same equipment, which was calibrated using identical methods, and were measured with identical laboratory techniques for the initial and the follow-up tests.

### Soccer training program

The training period was spread over 28 weeks (from October to April) (Table 2), where training occurred in four weekly sessions (between 5 pm and 6:15 pm on Tuesdays, Wednesdays, Fridays, and Sundays ) (Table 3). During this time, only friendly games were scheduled. Each training session included a 15 min warm-up period, followed by 20 min of physical work (jumps, sheathing, running) and 30 min of basic technical training (dribbling, juggling, passing, technical circuit), completed by active stretching exercises.

**Table 2:** The study design and sessions characteristics

Duration	Days Per Week	Total Sessions	Session Training Duration (min)	Repeated bout/frequency	Exercise bout/Recovery durantion	Work Intensity	Training Drills
28 weeks	4	135	75 mn	5–10	10-20s/10-20s	≥ 90%FCmax	Warm-up (15 min); acceleration running (5 min); technical soccer exercise (30 min); physical exercise (20 min) stretching (4 min);

**Table 3:** Exemplified microcycle of soccer training

Training week	Monday	Tuesday	Wednesday	Thursday	Friday	Saturday	Sunday
	Day off	- warm-up (15 min) - technical training (30 min) - low to moderate intensity aerobic exercises (20 min) - dynamic stretching	- warm-up (15 min) - technical training (30 min) - speed/coordination training (20 min) - small sided games	Day off	- warm-up (15 min) - soccer specific training (30 min) - tactical training (20 min) -reduced games	Day off	- match day (7vs.7) on small pitch (30 × 40 m).

The SG was exposed to seven months (i.e., 28 consecutive weeks) systematic soccer training with three sessions per week. The training sessions were performed on a synthetic soccer field and led by three professional coaches. During the first 6 weeks, coaches laid a focus on exercising physical fitness components such as endurance, linear and change-of-direction speed, coordination including balance to lay a foundation for subsequent soccer-specific training. From weeks 7 to 12, the exercise program primarily included soccer-specific technical drills (passing, dribbling, controlling the ball…).

From weeks 13 to 18, the coaches took care of development of motor skills such as gestural coordination, speed, joint flexibility, basic endurance through circuits, slaloms, races, jumps etc. From weeks 19 to 28, coaches focused on technical and tactical aspects of training including ball possession, recovering the ball, transition… and proposing simple situations of reduced squads by limiting the opposition (3 × 2; 4 × 3; 5 × 4…) in addition to fun games and small sided games (3 × 3; 4 × 4; 5 × 5 on small-medium pitches 12 × 20 m; 16 × 24 m; 25 × 35 m). The last 10 weeks were reserved for technical development, maintenance of physical qualities and tactical placement (mainly by player position). The model applied by the coaches followed the one proposed by the Tunisian Federation of Football.

Training intensity was regulated and controlled using smartwatches (H.Tang, Model F6, China) which made it possible to monitor the heart rate during continuous running (i.e., 50–70% of maximum heart rate [HRmax]), high-intensity interval training (i.e., 90–100% of HRmax), specific soccer training with and without a ball, tactical training, technical training, small sided games, and aerobic training. Moreover, a soccer game was scheduled each week on Sunday. The participating team played other regional clubs, using a 7 × 7 format on a relatively small pitch (30 ≠ 40 m). The game lasted 15 min during each halftime. SG played 20 matches during the experiment period.

The participating boys were part of the same soccer training center in the city of Sousse (Tunisia). Nine out of 40 children practiced soccer in a private training center for two years (at most) prior to the start of the study. All other participants started with playing soccer as they entered the study. During the same period, CG followed a physical education course in their primary school at the rate of one-hour session per week, the content of which was fundamentally playful based on educational games. Boys did not participate in any other physical training activities during the study. The children in both groups completed all aspects of the training programs. The exercise training attendance rate for the experimental group (e.g., soccer group) was 91%. No test or training-related injuries occurred during the study.

### Statistical analyses

All results are presented as means and standard deviations (SDs) after the normality distribution of data was assessed and confirmed using the Shapiro–Wilk test. The intraclass correlation coefficient (ICC) and the coefficients of variation (CV) were used to determine the consistency of the measures and their variation (test-retest reliability). Based on the 95% CI of the ICC estimate, values less than 0.5, between 0.5 and 0.75, between 0.75 and 0.9, and greater than 0.90 were indicative of poor, moderate, good, and excellent agreement, respectively. Within and between-group differences were calculated using a two-way analysis of variance (ANOVA) for repeated measures. Bonferroni adjusted post hoc tests were calculated to assess any significant group-by-time interactions. Partial eta squared (η^2^p) were taken from ANOVA output, and Cohen’s d effect sizes (d) were calculated to quantify meaningful differences in the data [24, 25] with demarcations of trivial (< 0.2), small (0.2–0.59), medium (0.60–1.19), large (1.2–1.99), and very large (≥ 2.0).

Pearson correlations were used to examine the relationship between variables. The magnitude of the correlations was determined using the modified scale prposed by Hopkins (2009) [26]. A step-wise multiple regression analysis was used to determine the best predictor independent variables. We tried to use a stepwise regression between the VO_2_max, MAP) and the pulmonary parameters (TL_NO_, TL_CO_, VA, Vc, DM).

All statistical analyses were computed using SPSS for Windows, version 16.0 (SPSS Inc., Chicago, IL, USA).

## Results

No statistically significant baseline between-group differences were observed for any of the assessed variables (p > 0.05 as shown in Table 1. Our results have a high test–retest reliability, with interclass correlation coefficients (ICCs) of 0.94 for DM and an ICC of 0.87 for TLCO. The variations in pulmonary and functional parameters at rest and just at the end of maximal effort before and after the 28 weeks are summarized in Tables S4 and S5.

### Main effects of time

#### At rest

**Fig. 1:**
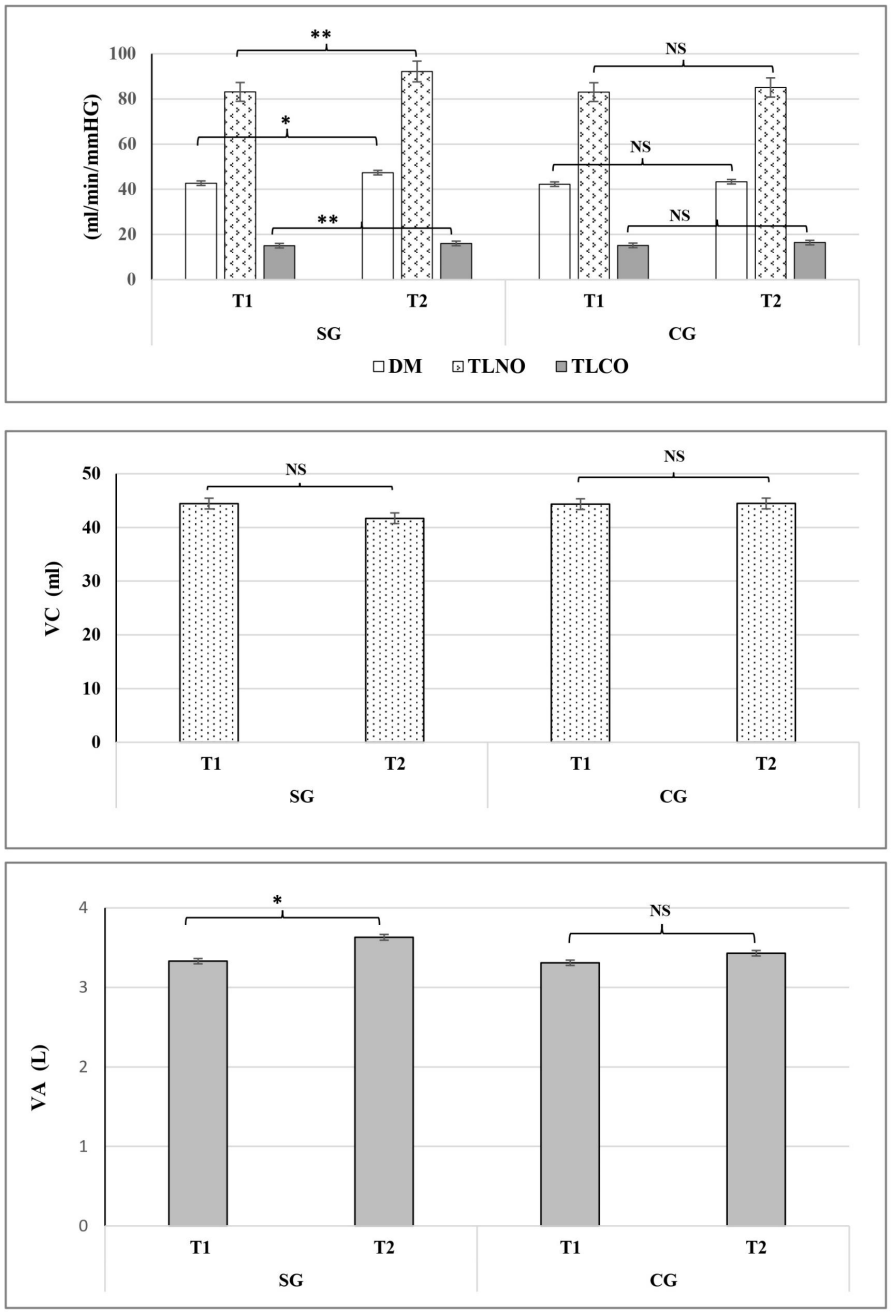
Mean (± SD) Pulmonary parameters [membrane factor for CO (DM_CO_), nitric oxide lung transfer (TL_NO_) and carbon monoxide lung transfer (TL_CO_), lung capillary blood volume (Vc) and alveolar volume (VA)], measured at rest before (T1) and after the training period (T2) in soccer (SG) and control groups(CG). NS: no significant difference. *: Significant difference: *p˂0.05 and **p˂0.01

Resting heart rate and most pulmonary parameters displayed significant main effects of time (post-test > pre-test, p < 0.05), except for Vc (p = 0.173, ES = 0.012). The variations of the ES ranged from small to large for all studied parameters (0.2 < ES < 4.0). However, it should be noted that changes primarily occurred in DM and TL_NO_ (~ 13%) in the SG (Table S4). Significant improvements were identified for DM (p < 0.05), TL_NO (_p < 0.01), TL_CO_ (p < 0.01), and VA (p < 0.05) in the SG (Fig. 1).

#### After the maximal exercise test

**Fig. 2:**
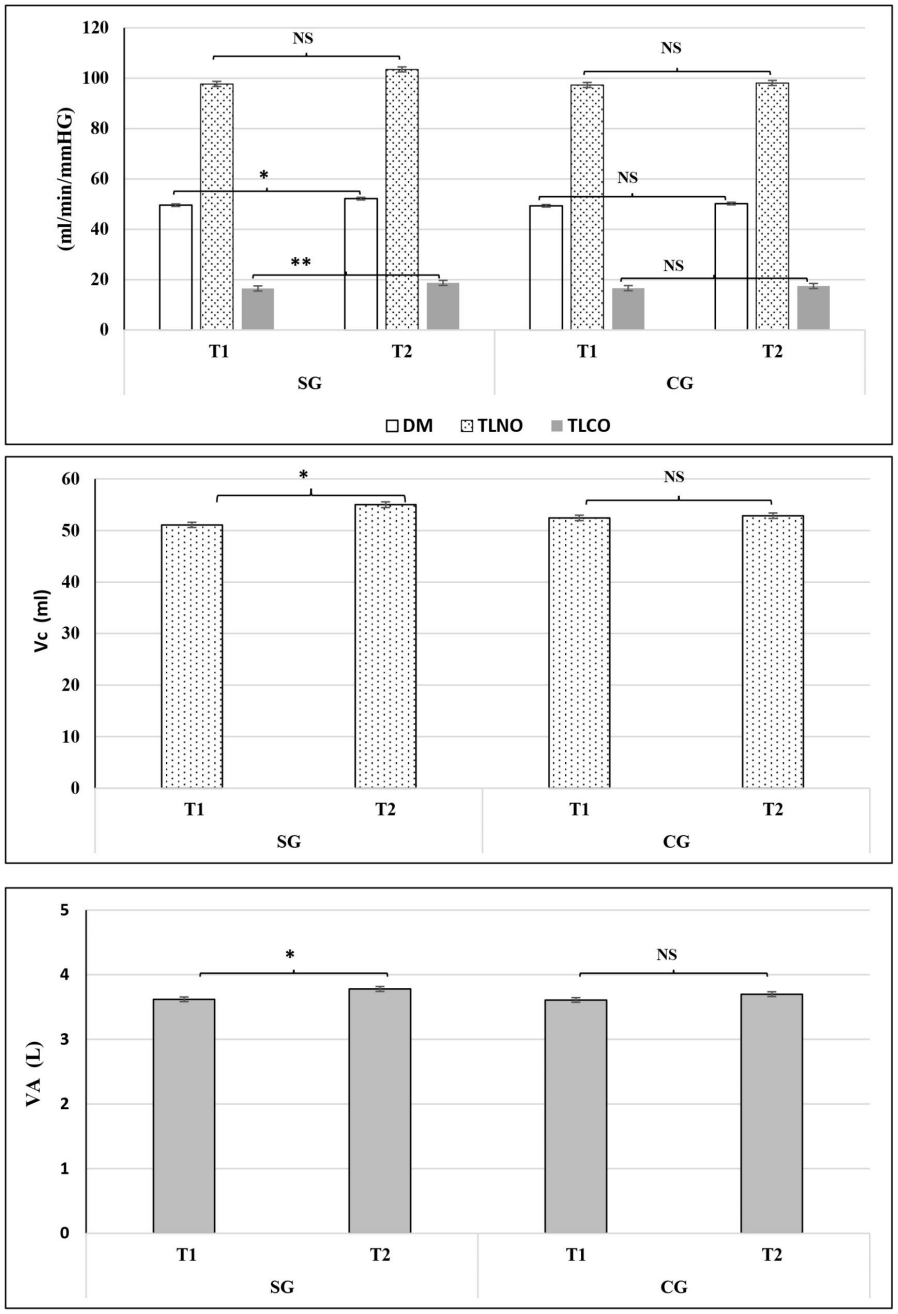
Mean (± SD) Pulmonary parameters [membrane factor for CO (DM_CO_), nitric oxide lung transfer (TL_NO_) and carbon monoxide lung transfer (TL_CO_), lung capillary blood volume (Vc) and alveolar volume (VA)], measured just afte maximal exercise before (T1) and after the training period (T2) in soccer (SG) and control groups (CG). NS: no significant difference. *: Significant difference: *p˂0.05 and **p˂0.01

Significant main effects of time were noted for maximal heart rate and VO_2max_ (p = 0.001, ES = 0.44; p = 0.002; ES = 0.26), respectively. The ES variations ranged from small to large for the assessed parameters (0.2 < ES < 4.0). Changes were noted for the majority of the assessed pulmonary parameters measured at the end of the maximal exercise test (Table S5). Values for TL_CO_ improved by 20%; Vc by 14% DM by 8%, and VA by 10% at the end of the maximal exercise in the SG (Table S5). Furthermore, the observed changes were smaller in the control group (7.5% for TL_CO_; 2% for Vc; 5% for DM, 4% for VA). Moreover, there were significant increases (p < 0.05) in total pulmonary parameters (from T_1_ to T_2_) only in the SG (Fig. 2**).**”

### Group-by-time interactions

#### At rest

Most pulmonary parameters measured at rest showed significant group by time interactions (p < 0.05), with ES values ranging from small to large for most parameters (0.2 < ES < 4.0), except for VA (p = 0.3, ES = 0.006) (Table S4). Post-hoc tests indicated significant improvements for DM (p < 0.05; 0.2 < ES < 4.0), TL_NO_ (p < 0.01; 0.22 < ES < 4.0), TL_CO_ (p < 0, 01; 0.24 < ES < 4.0) and Vc (p = 0.01; 0.404 < ES < 0.6) in SG but not CG.

#### After the maximal exercise test

Significant group by time effects were found for HRmax, VO_2_max, and MAP (p < 0.001; ES = 0.5; p = 0.005; ES = 0.23 and p = 0.04; ES = 0.25 respectively) (Table 1). The post-hoc analyses indicated a significant decrease in HRmax and a significant increase in VO_2_max in the SG but not the CG (p < 0.001; ES = 0.5 and p = 0.05, ES = 0.023 respectively). There were significant group by time interactions (p < 0.05) for pulmonary parameters measured at the end of the maximal exercise test. The ES magnitudes ranged from small to large for all parameters (0.2 < ES < 4.0). The post hoc analysis indicated significant improvements for DM (p = 0.003, ES = 0.491), TL_NO_ (p = 0.008, ES = 0.261), TL_CO_ (p = 0.013, ES = 0.55), and Vc (p < 0.01, ES = 0.39) in SG but not CG (Table S5).

Stepwise linear regression analyses were calculated to identify the relation between VO_2max_ and MAP and pulmonary diffusion parameters (TL_NO_, TLCO, VA, Vc, DM). No significant correlations were found for all analyzed parameters (Table S6).

## Discussion

This study analyzed the effect of 28 weeks of regular soccer training versus control on lung diffusion capacity and its components in prepubertal boys. The main findings showed significant group by time interactions (p < 0.05) after soccer training for most pulmonary parameters measured at rest. For SG but not CG, post hoc tests indicated significant improvements for DM, TL_NO_, TL_CO,_ and Vc after the maximal exercise test. Moreover, significant group by time effects were identified for HRmax, VO_2_max, and MAP and pulmonary parameters (DM, TL_NO_, TL_CO,_ and Vc) measured at the end of the maximal exercise test in favor of SG.

The improvement in pulmonary function parameters observed after 28 weeks of regular soccer training could be related to an increased permeability of the alveolar-capillary membrane. Previous studies showed an improved pulmonary capillary bed distensibility in young basket-ball players and middle distance runners [9, 10, 27]. However, intense exercise can increase ventilation and induce mechanical stress between the lung and thorax [28], while increased ventilation and pulmonary blood flow during physical exertion could adjust capillary permeability and support the integrity of the alveolar-capillary barrier [28–30].

### Soccer training and performance development

The effect of regular soccer training on performance development has been reported by other researchers [10, 31]. Our study confirmed that regular sports practice improved VO_2_max which was associated with a decrease in HRmax, which can be attributed in part to the hypervolemia induced by increases in cardiac stroke volume due to regular training [32]. The differences in maximal oxygen consumption (3%) between the two groups of children (SG and CG) in our study are similar to reports by others [33]. Moreover, there was an improvement in MAP and we acknowledge that in children, mostly pre-pubescent, the MAP improvement requires repeated and relatively intense muscular efforts to induce lasting functional changes. Along with this, the improvement in VO_2_max through systematic training of the aerobic system is undeniable. Moreover, the development of the MAP is generally developed by the repetition of intermittent exercises with high intensity [34], and this is what characterizes the efforts in soccer. Gerbeau et al. specified that the progress (often very controversial) of VO_2_ max and MAP in trained children depends on the nature and duration of the cycles [35].

We performed a stepwise linear regression analysis to identify potential associations between VO_2_max and MAP and pulmonary diffusion parameters (TL_NO_, TL_CO_, VA, Vc, DM). No significant correlations were found between the analyzed parameters. Our results confirmed the findings of a previous study which was able to show that regular soccer training has the potential to improve aerobic capacity and VO_2_max [5]. However, no statistically significant associations were found between deltas of performance and pulmonary diffusion capacity parameters.

### Soccer practice and lung function

The resting pulmonary parameters of our population are similar to those reported in the literature [10]. The increases in these volumes from rest to exercise in both groups of children were attributed to a greater inspiratory impulse that results in improved contraction of the respiratory muscles and changes in lung mechanics induced by exertion [18].

The ratio of Vc/VA at rest in both groups of children at T_2_ was approximately 13 ml.L^− 1^, a value close to that reported for a similar population of children [10] or for young adults [36]. This suggests that the growth of pulmonary capillaries parallels the growth of the lung parenchyma, especially between the age of ten and adulthood. Moreover, Vc/VA increased in both groups of children (14 ml.L^− 1^) just after stopping exercise, which indicates a possible implication of cardiac blood flow in the dispersion of Vc values ​​at the end of maximal effort; in fact, the increase of Vc has been attributed to the recruitment of capillaries as well as their increase in diameters [28, 37]. However, DM and Vc correlated with cardiac blood flow (Q’c), as physical exercise increases pulmonary arterial pressure (Ppa) as well as pulmonary capillary pressure (Pcap) to increase Vc by recruitment and distension of capillaries [10]. However, DM and VA increased significantly from rest to exercise in both groups. Since DM_CO_ was derived directly from TL_NO_, and as NO is highly reactive with hemoglobin, as such its transfer is almost independent of Vc but strongly dependent on membrane properties [9].

DM is a function of membrane thickness [38] and the increase in cardiac blood flow could decrease the thickness of the plasma layer near the capillary wall, which could increase the thickness of the alveolar-capillary membrane [9, 38], but which will occur in both groups of children and could not explain the difference between the trained and untrained children. This suggests that another hypothesis could be valid, where the alveolar-capillary membrane of soccer players was subjected to greater stretch (due to their greater maximum ventilation). In fact, intermittent physical exercise increases the strength of the respiratory muscles [11] and increases lung surface area [39], as shown in adults [37, 40] and children [10, 38]. It has been argued that the distribution of blood in pulmonary capillaries is more uniform in a population of trained boys compared to non-trained subjects, leading to an increased Vc value in response to a larger exchange surface [9]. Moreover, we can say that regular soccer training could be considered as a means of improving the diffusion capacity and pulmonary rehabilitation since it acts beneficially on the behavior of the pulmonary capillary bed.

The maximum workload (as assessed by MAP) was increased by 11.5%, which was associated with a slight improvement in VO_2_max (3.5%) in the soccer group after 28 weeks of training, suggesting that the respiratory muscles were more efficient at extracting oxygen [41], probably due to a better distribution of blood flow in the capillaries [42]. Our findings are in agreement with previous research associating VO_2_max and TL_CO_ [43] as well as pulmonary hemodynamics [43, 44].

## Limitations

There are some limitations to our study that should be acknowledged. First, we enrolled boys only in our study and cannot translate our findings to girls. Future studies should examine whether our findings can be replicated in prepubertal girls. Second, we were unable to examine additional factors (e.g., the thickness of the alveolar-capillary membrane, distribution of blood in pulmonary capillaries…). Third, the extracurricular sports activities were not monitored in the control group which prevents us from comparing the actual training volumes between groups. Nevertheless, the CG group was asked not to take up any new sports or physical activities during the time of the study. Moreover, the training load of the soccer group should be monitored in future studies using an accurate Global Positioning System (GPS).

## Conclusions

Our study indicates that regular soccer training improves pulmonary vascular function and increases DM and Vc immediately after the maximal exercise test in prepubertal boys. This finding can most likely by explained by better recruitment of pulmonary capillaries during physical exercise. Moreover, the stepwise linear regression analyses proved that increases in pulmonary vascular function not related with the observed improvements in VO_2_max and MAP. Furthermore, and although the exact mechanisms by which sports training improves pulmonary diffusion are not well-understood, it is likely that increases in the lung surface area for gas exchange and/or alterations in the thickness of the alveolar membrane mediates improves the function of the pulmonary capillary bed.

### Electronic supplementary material

Below is the link to the electronic supplementary material.

Supplementary Material 1

## Data Availability

The datasets generated during and analyzed during the current study are not publicly available due to confidential information about the participants but are available from the corresponding author on reasonable request.

## Abbreviations

CG: Control group
CO: Carbon monoxide
DM: Membrane component of alveolar-capillary transfer of gases (mL.min^− i^.mmHg^− 1^)
DM_Co_: Membrane component of alveolar-capillary transfer of carbon monoxide (mL.min^− i^.mmHg^− 1^)
SG: Soccer group
HR_max_: Maximal heart rate
MAP: Maximal aerobic power
NO: Nitric oxide
rpm: Revolutions per minute
TL: Pulmonary diffusion capacity
TL_CO_: Pulmonary diffusion capacity for carbon monoxide (mL.min^− 1^. mmHg^− 1^)
TL_NO_: Pulmonary diffusion capacity for nitric oxide (mL.min^− 1^. mmHg^− 1^)
TL_NO/CO_: Carbon monoxide and nitric oxide transfer measurement
VA: Alveolar volume (L)
Vc: Capillary blood volume (mL)
VO_2max_: Maximal oxygen uptake
