# Supplementary material for: Regular soccer training improves pulmonary diffusion capacity in 6 to 10 year old boys
Source: BMC Sports Sci Med Rehabil. 2023 Nov 2;15:146. doi: 10.1186/s13102-023-00757-6 (PMC10621163; doi:10.1186/s13102-023-00757-6)
Supplement: Supplementary file 1 — Supplementary Material 1 [file 13102_2023_757_MOESM1_ESM.docx]

**Table S4**: Pulmonary parameters measured at rest: before and after the training period in soccer and control groups.

| **Parameters** | **Soccer training group (n=40)** | | | | **Control group (n=40)** | | | | **Analysis of variance (ANOVA) and effect sizes (eta-squared)** | | | | | |
| --- | --- | --- | --- | --- | --- | --- | --- | --- | --- | --- | --- | --- | --- | --- |
|  |  |  |  |  |  |  |  |  | **Group** | | **Time** | | **Group x Time** | |
|  | **pre** | **post** | **d** | **%** | **pre** | **post** | **d** | **%** | **p** | **η2** | **p** | **η2** | **p** | **η2** |
| **DM**  (ml/min/mmHg) | 42.71±6.24 | 47.38±5.96 | 0.77 | 12.85±1.87 | 42.28±8.08 | 43.35±8.75 | 0.13 | 6.08±1.81 | 0.06 | 0.02 | 0.01 | 0.04 | 0.01 | 0.20 |
| **TL_NO_**  (ml/min/mmHg) | 83.14±10.42 | 92.16±12.28 | 0.79 | 12.77±2.89 | 83.04±11.51 | 85.08±12.28 | 0.17 | 4.24±0.78 | 0.063 | 0.024 | 0.003 | 0.055 | 0.006 | 0.022 |
| **TL_CO_**  (ml/min/mmHg) | 15.04±2.43 | 16.05±1.62 | 0.49 | 8.66±1.49 | 15.18±2.69 | 16.40±2.28 | 0.48 | 11.35±1.42 | 0.510 | 0.003 | 0.002 | 0.057 | 0.007 | 0.024 |
| **Vc** (ml) | 44.45±6.08 | 41.71±2.08 | 0.60 | -4.36±1.60 | 44.34±6.44 | 44.46±7.95 | 0.10 | 2.46±1.35 | 0.170 | 0.012 | 0.173 | 0.012 | 0.01 | 0.404 |
| **VA** (L) | 3.33±0.66 | 3.63±0.46 | 0.53 | 11.64±1.17 | 3.31±0.71 | 3.43±0.54 | 0.19 | 7.67±1.01 | 0.26 | 0.008 | 0.029 | 0.030 | 0.30 | 0.006 |

*Data are mean ± standard deviation (SD). Values for interaction effects and effect sizes (ESs) are also shown. Data are mean ± standard deviation (SD) of alveolar volume (VA), nitric oxide lung transfer (TL_NO_), carbon monoxide lung transfer (TL_CO_), membrane factor for CO (DmCO) and lung capillary blood volume (Vc)*

**Table S5**: Pulmonary parameters measured after the maximal exercise: before and after the training period in soccer and control groups.

| **Parameters** | **Soccer training Group (n=40)** | | | | **Control Group (n=40)** | | | | **Analysis of variance (ANOVA) and effect sizes (eta-squared)** | | | | | |
| --- | --- | --- | --- | --- | --- | --- | --- | --- | --- | --- | --- | --- | --- | --- |
|  |  |  |  |  |  |  |  |  | **Group** | | **Time** | | **Group x Time** | |
|  | **pre** | **post** | **d** | **%** | **pre** | **post** | **d** | **%** | **p** | **η2** | **p** | **η2** | **p** | **η2** |
| **D_M_**  (ml/min/mmHg) | 49.57±8.23 | 52.17±6.89 | 0.34 | 8.23±2.78 | 49.33±8.51 | 50.22±7.57 | 0.11 | 5.20±2.76 | 0.005 | 0.377 | 0.016 | 0.013 | 0.003 | 0.491 |
| **TL_NO_**  (ml/min/mmHg) | 97.72±12.77 | 103.48±15.69 | 0.40 | 7.78±2.35 | 97.31±12.85 | 98.11±14.01 | 0.06 | 3.72±1.77 | 0.011 | 0.190 | 0.137 | 0.140 | 0.008 | 0.261 |
| **TL_CO_**  (ml/min/mmHg) | 16.50±3.49 | 18.70±3.52 | 0.63 | 19.51±1.30 | 16.63±2.58 | 17.48±2.06 | 0.36 | 7.56±2.05 | 0.008 | 0.252 | 0.001 | 0.063 | 0.013 | 0.55 |
| **Vc** (ml) | 51.08±12.60 | 55.28±14.04 | 0.31 | 14.21±1.45 | 52.44±6.88 | 52.86±12.01 | 0.04 | 2.34±0.95 | 0.001 | 0.772 | 0.021 | 0.010 | 0.007 | 0.39 |
| **VA** (L) | 3.62±0.75 | 3.78±0.55 | 0.24 | 9.82±1.22 | 3.61±0.45 | 3.70±0.50 | 0.19 | 3.95±1.39 | 0.001 | 0.636 | 0.016 | 0.012 | 0.001 | 0.728 |

*Data are mean ± standard deviation (SD) of alveolar volume (VA), nitric oxide lung transfer (TL_NO_), carbon monoxide lung transfer (TL_CO_), membrane factor for CO (DM_CO_), and lung capillary blood volume (Vc).*

**Table S6:** Correlation coefficients between functional parameters (MAP, VO_2_max) and pulmonary diffusing capacity parameters of the soccer Group (n=40).

|  | **MAP**  (Watts) | **VO_2_max**  (ml·min^-1^·kg^-1^) | **VA**  (L) | **TL_NO_**  (mL·min^−1^ ·mmHg^−1^) | **TL_CO_**  (mL·min^−1^ ·mmHg^−1^) | **DM**  (mL·min^−1^ ·mmHg^−1^) | **Vc**  (mL) |
| --- | --- | --- | --- | --- | --- | --- | --- |
| **MAP**  (Watts) | 1 | 0.213 | -0.045 | -0.251 | 0.11 | 0.187 | -0.003 |
| **VO_2_max**  (ml·min^-1^·kg^-1^) | 0.213 | 1 | -0.106 | -0.241 | -0.050 | 0.195 | -0.16 |
| **VA**  (L) | -0.045 | -0.106 | 1 | 0.261 | 0.691**^**^** | 0.032 | 0.626**^**^** |
| **TL_NO_**  (mL·min^−1^ ·mmHg^−1^) | -0.251 | -0.241 | 0.261 | 1 | 0.223 | 0.128 | 0.218 |
| **TL_CO_**  (mL·min^−1^ ·mmHg^−1^) | 0.011 | -0.050 | 0.691**^**^** | 0.223 | 1 | -0.014 | 0.833**^**^** |
| **DM**  (mL·min^−1^ ·mmHg^−1^) | 0.187 | 0.195 | 0.032 | 0.128 | -0.14 | 1 | -0.112 |
| **Vc**  (mL) | -0.003 | -0.16 | 0.626**^**^** | 0.218 | 0.833 | -0.112 | 1 |

**^**^**Correlation is significant at the 0.01 level (2-tailed)

**MAP**: maximal aerobic power (Watts); **VO_2_max:** maximal oxygen uptake (ml·min^-1^·kg^-1^); **DM**: membrane component of alveolar-capillary transfer of gases (mL^.^min^-^1.mmHg^-1^); **TL_CO_**: pulmonary diffusion capacity for carbon monoxide (mL^.^min^-1.^ mmHg^-1^); **TL_NO:_** pulmonary diffusion capacity for nitric oxide (mL^.^min^-1.^ mmHg^-1^); **VA:** alveolar volume (L); **Vc:** capillary blood volume (mL).
